# Punish the thief—coevolution of defense and cautiousness stabilizes ownership

**DOI:** 10.1007/s00265-017-2330-4

**Published:** 2017-06-16

**Authors:** Martin Hinsch, Jan Komdeur

**Affiliations:** 1 Imperial College London, South Kensington Campus, London, SW7 2AZ UK; 2 57 Agar Crescent, Bracknell, RG42 2BY UK; 3 University of Groningen, GELIFES, Nijenborgh 7, 9747 AG Groningen, The Netherlands

**Keywords:** Resource defense, Ownership, Deterrence, Punishment, Territoriality

## Abstract

**Abstract:**

Ownership of non-controllable resources usually has to be maintained by costly defense against competitors. Whether defense and thus ownership pays in terms of fitness depends on its effectiveness in preventing theft. We show that if the owners’ willingness to defend varies in the population and information about it is available to potential thieves then the ability to react to this information and thus avoid being attacked by the owner is selected for. This can lead to a positive evolutionary feedback between cautiousness in intruders and aggressiveness in owners. This feedback can maintain ownership when the actual direct effectiveness of defense in reducing theft is very low or even absent, effectively turning defense into punishment. We conclude that the deterrence effect of defense in many situations could be stronger than that of prevention and that for many real-world scenarios the purpose of defense of resources might be to punish rather than to drive away intruders.

**Significance statement:**

Many animals defend resources against conspecifics. Resource defense can usually only evolve if its costs are paid for by foiling attempts at theft. We show that if potential thieves can detect differences in aggressiveness between owners then cautious intruders and aggressive owners coevolve so that in the end even ineffective defense deters thieves and maintains ownership. This result greatly extends the number of situations in which we expect resource defense to evolve and has the potential to unify the concepts of defense and punishment.

## Introduction

Competition for resources such as food or mates is ubiquitous in animals. In order to be able to profit from a resource, an individual therefore has to keep competitors away from it (Strassmann and Queller 2014). Some resources such as small food items can be consumed immediately so that access by conspecifics is easily prevented. For others such as territories or mates, ownership has to be established by means of defense, that is, an aggressive action that reduces a competitor’s access to the defended item (Brown 1964; Maynard Smith and Price 1973; Hinsch et al. 2013).

Defense is usually costly in terms of time, energy, or risk of injury (Schoener 1983). Whether ownership of a given type of resource is viable in a population therefore depends on whether defense confers a fitness advantage, i.e., whether these costs are lower than the benefit of increased exclusiveness of access to the resource (Brown 1964). Whether it pays in terms of fitness for the prospective thief to attempt theft depends in turn on the likelihood of being attacked by the owner and the costs of the potentially ensuing fight (Dubois and Giraldeau 2005). If fighting costs are high and defense (i.e., attack by the owner) is likely enough, therefore, theft can become entirely unprofitable (Hinsch and Komdeur 2010). This deterrence effect, however, takes place solely in evolutionary time and therefore affects the evolution of defense only indirectly by reducing the average level of attempts of theft and thus the costs of defense.

From studies on the evolution of cooperation, we know that deterrence that instead works on the individual time scale can have much more significant effects: If an individual’s tendency to punish non-cooperators is known to its competitors before they interact with it, they can adjust by being more cooperative towards eager punishers which in turn causes selection for increased punishment. This feedback can be strong enough to lead to the evolution of full “altruistic” cooperation (Johnstone 2001; dos Santos et al. 2010; Schoenmakers et al. 2014). A similar deterrence effect of aggression has been postulated for dominance hierarchies (Thompson et al. 2014).

Already, Stamps (1994) and Stamps and Krishnan (1999) suspected that individual-level deterrence could have similarly dramatic effects on the evolution of resource defense. They even suggested that the main function of aggression towards intruders on territories might be to deter them from intruding and stealing resources again rather than to actively chase them away. It has indeed been shown that if aggression towards intruders is assumed to reduce repeat intrusions, defense by owners is strongly selected for (Switzer et al. 2001). On the other hand, we know that it can generally be adaptive for individuals to avoid returning to the location of a costly encounter (Morrell and Kokko 2003, 2005). It can therefore be suspected that in a similar way to the coevolution of punishment and cooperation, sensitivity of intruders for the owner’s aggressiveness and willingness to defend could coevolve to the point where ownership of resources becomes established.

In this study, we investigate how the establishment of ownership of resources by coevolution of defense and theft is affected if potential thieves can obtain and react to information about an owner’s willingness to defend. Relative to previous models, we make two additional assumptions. First, potential thieves either know or are able to learn by experience the owner’s aggressiveness. Second, intruders can evolve sensitivity, i.e., the ability to adjust their tendency to steal from a particular owner based on perceived aggressiveness.

We first introduce a simple mathematical model which allows for a clear demonstration of the general mechanisms involved. In order to verify our results in a more realistic setting, we also investigate a more detailed individual-based simulation of a population of territory owners and floaters competing for resources within territories.

It is important to note that we do not model a particular species and that the floater/territory owner scenario we use for the simulation model is only meant to serve as an example. The evolutionary mechanism we propose is general enough that it could potentially occur in many different real-world situations where animals defend some kind of property against competitors as long as it is in principle shareable (note that that excludes for example defense of entire territories, see Hinsch and Komdeur 2017) such as defense of carcasses or mate guarding (van Lieshout and Elgar 2011; Rousseu et al. 2014; Sherratt and Mesterton-Gibbons 2015).

## Analytical argument

We will first use a simple conceptual model to show under which conditions there is selection for defense and sensitivity, respectively.

As our base model, we choose a basic sequential move game representing the encounter between an owner and a thief or intruder. The model is structurally similar to others used previously to investigate conflicts between owners and intruders (e.g., Dubois et al. 2003; Hinsch and Komdeur 2010; Eswaran and Neary 2014). For the sake of simplicity, we only model the actual interactions between individuals, leaving out for now details such as resource distribution, search time, and frequency of owners and non-owners.

An interaction begins with the intruder deciding whether to attempt theft. If it does not, it has to search for resources elsewhere which we assume results in a frequency-dependent (due to competition with other searchers) payoff of $P\left(\widehat{t}\right)$, with $\widehat{t}$ as mean population tendency to steal. In this case, the owner will gain the full amount *V*. If the intruder does attempt to steal resources, the owner decides whether to defend or not (following its aggressiveness *a*). If the owner concedes, the intruder will steal an amount *T* of resources leading to a reduced payoff of *V* − *T* for the owner. If the owner does defend, the intruder only manages to steal a small amount of *S* (*S* < *T*) resources; however, both intruder and owner have to pay fighting costs *C* leading to payoffs *S* − *C*_*t*_ and *V* − *S* − *C*_*o*_, respectively. Table 1 shows the resulting payoffs for all combinations of actions.

**Table 1:** Payoffs in the basic model for owner and intruder, respectively

Intruder		*Steal*		*Stay away*
Owner				
		*S* − *C*_*t*_		$P\left(\widehat{t}\right)$
*Defend*	*V* − *C*_*o*_ − *S*		*V*	
		*T*		$P\left(\widehat{t}\right)$
*Concede*	*V* − *T*		*V*	

### No sensitivity, no variation

Strategies in the basic model—tendency to attempt theft *t* for the intruder and aggressiveness *a* for the owner—are modeled as simple probabilities. Given these strategies, we can easily spell out the expected payoffs *w* for a rare mutant intruder (*t*) and owner (*a*), respectively, in a homogeneous resident population ($\widehat{t}$ and $\widehat{a}$):

1 $$ \begin{array}{ccc}{w}_t(t)& =& \left(1- t\right) P\left(\widehat{t}\right)+ t\left(\left(1-\widehat{a}\right) T+\widehat{a}\left( S-{C}_t\right)\right)\\ {}{w}_o(a)& =& \left(1-\widehat{t}\right) V+\widehat{t}\left(\left(1- a\right)\left( V- T\right)+ a\left( V-{C}_o- S\right)\right)\end{array} $$

We determine the direction of selection by calculating the gradient of the mutant’s fitness around the resident’s trait value as ${\left.{w_t}^{\prime }= d{w}_t(t)/ dt\right|}_{t=\widehat{t}}$ and ${\left.{w_o}^{\prime }= d{w}_o(a)/ da\right|}_{a=\widehat{a}}$. An increase in trait value is selected for if the respective derivative is positive (Geritz et al. 1998).

For selection on *a*, we obtain

2 $$ {w_o}^{\prime }=\widehat{t}\left( T-{C}_o- S\right) $$

It follows that as long as there is any theft ($\widehat{t}>0$), aggressiveness increases if the cost of defense is lower than the benefit of preventing theft:

3 $$ T- S>{C}_o $$

In particular, if defense has no direct prevention effect (*T* = *S*), then it can never be selected for.

For the intruders, we find

4 $$ {w_t}^{\prime }=- P(t)+\left(1- a\right) T+ a\left( S-{C}_t\right) $$

The propensity to steal correspondingly increases if the net benefit of stealing is greater than the benefit of the outside option (searching for resources elsewhere):

5 $$ \left(1- a\right) T+ a\left( S-{C}_t\right)> P(t) $$

Since the fitness benefits of aggressiveness are not frequency dependent in this model, we will always end up with pure strategies for *a*, i.e., either “always attack” (*a* = 1) or “never attack” (*a* = 0).

For these, we can further simplify inequality 5 to either *T* > *P*(*t*) (for *a* ^∗^ = 0) or *S* − *C*_*t*_ > *P*(*t*) (for *a* ^∗^ = 1). Depending on the choice of function *P*, pure strategies for *t* are therefore possible if the benefit of the outside option is always lower or always higher than the benefit of stealing irrespective of the frequency of theft. For the purpose of a clear demonstration of the mechanisms involved, we will, however, restrict our discussion in the following to the more relevant cases where *t* has inner equilibria (i.e., 0 < *t* ^∗^ < 1).

### Deterrence selects for aggressiveness

In the next step, we investigate the effect of sensitivity for aggressiveness on the evolution of defense. We assume that intruders have a way of knowing the aggressiveness of an owner they are about to interact with in advance (e.g., by observing conflicts with others or by experience) and are able to modify their behavior accordingly. Therefore, instead of by a fixed probability *t* to make an attempt at theft, the intruders’ behavior is now determined by a sensitivity function *t*(*a*) that depends on an owner’s aggressiveness *a*.

We will first investigate how *a* evolves dependent on the properties of a given (unspecified) function *t*. Only after that will we take a closer look at the evolution of *t* itself.

Apart from the change in notation, payoffs remain the same as before (see Eq. 1):

6 $$ {w}_o(a)=\left(1- t(a)\right) V+ t(a)\left(\left(1- a\right)\left( V- T\right)+ a\left( V-{C}_o- S\right)\right) $$

which gives us the following selection gradient (with ${t}^{\prime }:=\frac{dt(a)}{da}$, *w*′_*o*_ : = *dw*_*o*_(*a*)/*da*):

7 $$ {w}_o^{\prime }= t( a)\left( T-{C}_o- S\right)-{t}^{\prime}\left(\left(1- a\right) T+ a\left( S+{C}_o\right)\right) $$

As before, this equation has a straightforward interpretation. The first term on the right hand side is identical (with *t* replaced by *t*(*a*)) to the selection gradient in the simple model (Eq. 2) and therefore represents the direct benefits of defense.

In addition, however, as soon as intruders are responsive to the owner’s aggressiveness (i.e., as soon as *t* ^′^ ≠ 0), there is now a second term representing an additional indirect effect of aggressiveness. If intruders are cautious (*t* ^′^ < 0), this term becomes positive and even increases with *a* if defense is costly (*S* + *C*_*o*_ > *T*). This deterrence effect can thus provide an additional strong benefit to aggressiveness.

For the situation without any direct benefits of defense (*T* = *S*) that always lead to the disappearance of aggressiveness in the simple model, we obtain now

8 $$ {w_o}^{\prime }=- t(a){C}_o-{t}^{\prime}\left( T+ a{C}_o\right) $$

If deterrence is strong enough (i.e., intruders are cautious enough), defense can therefore be selected for even if it has no effect on the amount stolen at all.

### Variation selects for sensitivity

In the last step, we take a look at the evolution of sensitivity, i.e., the ability of intruders to adjust their behavior. Behavioral flexibility can be costly (e.g., Auld et al. 2010; Burns et al. 2011); therefore, we do not expect it to evolve in an entirely homogeneous population of owners where sensitivity has no benefit for the intruders at all. In most real populations, however, a trait like aggressiveness will show some variation due to, e.g., mutation, developmental effects such as age or condition, or differences in personality (McNamara et al. 2008; Wolf et al. 2008). It might therefore be advantageous for intruders to be able to adjust their behavior despite additional costs.

As before, we assume that intruders know (by experience or observation) how likely they are going to be attacked when stealing from a specific owner. Let *t*(*a*) again denote the probability that an intruder steals dependent on the owner’s aggressiveness.

We model variation in *a* by assuming that with probability *p*_*i*_ intruders will encounter an owner with aggressiveness *a*_*i*_. For a given population of owners, we can calculate the benefit of not intruding from the expected probability to steal $E\widehat{t}$ as $\widehat{P}:= P\left( E\widehat{t}\right)$. The cost of sensitivity *c*_*s*_ we assume to be proportional to the variance in *t*:

9 $$ {c}_s(t)={C}_s\mathrm{VAR}\left( t\left({a}_i\right)\right) $$

Defining the expected benefit of intrusion

10 $$ {I}_i:=\left(1-{a}_i\right) T+{a}_i\left( S-{C}_t\right), $$

we can now formulate our fitness function for the thief:

11 $$ {w}_t=-{c}_s(t)+\sum {p}_i\left[\left(1- t\left({a}_i\right)\right)\widehat{P}+ t\left({a}_i\right){I}_i\right] $$

Since the trait value *t* is a function or a vector, calculating the selection gradient as we did it before is not easily possible. Instead, in order to determine the direction of selection, we determine the fitness of a single mutant *t* in a resident population $\widehat{t}$ that is assumed to be sufficiently large and homogeneous with respect to the trait under consideration. If the mutant’s fitness is positive, there is selection for, if it is negative, against that particular mutation. A trait value is evolutionarily stable if no mutant with a higher fitness exists.

For a given population of owners with aggressiveness values *a*_*i*_, we can write the mutant’s trait values as $t\left({a}_i\right)=\widehat{t}\left({a}_i\right)+{s}_i$. Substituting this into Eq. 11 (see Appendix), the difference between the mutant’s and the residents’ fitness $\varDelta {w}_t={w}_t-{w}_{\widehat{t}}$ then derives as

12 $$ \varDelta {w}_t=-\varDelta {c}_s+\sum {p}_i{s}_i\left({I}_i-\widehat{P}\right). $$

As we show in the Appendix for small mutation step sizes (and, for the sake of brevity, using *t*_*i*_ ≔ *t*(*a*_*i*_)), this can be rewritten as

13 $$ \varDelta {w}_t=\sum {p}_i{s}_i\left({I}_i-\widehat{P}-2{C}_s\left({\widehat{t}}_i- E\widehat{t}\right)\right). $$

From this, we can show (for details see Appendix) that there is a single evolutionarily (and convergence) stable strategy in this system that is given by

14 $$ \begin{array}{cc}\hfill {t}_i^{\ast }=\hfill & \hfill \frac{I_i-\mathrm{EI}}{2{C}_s}+{P}^{-1}\left(\mathrm{EI}\right)\hfill \\ {}\hfill =\hfill & \hfill \frac{\left(1-{a}_i\right) T+{a}_i\left( S-{C}_t\right)-\mathrm{EI}}{2{C}_s}+{P}^{-1}\left(\mathrm{EI}\right).\hfill \end{array} $$

As we see, in the stable state the probability to steal *t*_*i*_ is proportional to how much the fitness payoff from intruding into a territory that is defended with probability *a*_*i*_ differs from the average payoff for all territories. Since we can assume that fighting is costly (*C*_*t*_ > 0) and that an intruder that does not get attacked by the owner will always forage at least as much as an intruder that meets resistance (*T* ≥ *S*), *t*_*i*_ decreases linearly with *a* and is therefore directly proportional to the probability not to get attacked by the owner 1 − *a*_*i*_. Any variation in defense *a* therefore leads to the evolution of an equivalent variation in theft *t* and thus to the evolution of sensitivity (*t* ^′^ ≠ 0) and in particular cautiousness (*t* ^′^ < 0).

### Coevolution of cautiousness and aggressiveness

As we have shown, variation in aggressiveness leads to the evolution of cautiousness and cautiousness in turn increases selection for aggressiveness under certain conditions (see Eq. 7). Taking both results together, we can now predict that any variation in aggressiveness will indirectly produce additional selection pressure towards higher aggressiveness.

While a full analysis including all possible causes of variation in *a* would go beyond the scope of this paper, we will in the following show how developmental or condition effects can lead to a coevolution of cautiousness and aggressiveness. Let us assume that for a given trait value *a* developmental effects, aging, or individual condition leads to a distribution of phenotypes *a*_*i*_ = *a* + *d*_*i*_ with probability distribution *p*(*a*_*i*_) = *p*_*i*_. As before, the fitness of an individual with phenotype *a*_*i*_ is then (see Eq. 6)

15 $$ {w}_o\left({a}_i\right)=\left(1- t\left({a}_i\right)\right) V+ t\left({a}_i\right)\left(\left(1-\left({a}_i\right)\right)\left( V- T\right)+\left({a}_i\right)\left( V-{C}_o- S\right)\right). $$

The fitness of a *genotype a*, however, now has to be calculated as the expected fitness of its phenotypes:

16 $$ \begin{array}{ccc}\hfill {\overline{w}}_o(a)\hfill & \hfill \coloneq \hfill & \hfill \sum {p}_i{w}_o\left({a}_i\right)\hfill \\ {}\hfill \hfill & \hfill =\hfill & \hfill \sum {p}_i{w}_o\left( a+{d}_i\right)\hfill \end{array} $$

From this, we can obtain the selection gradient with respect to *a* in exactly the same way as we did it previously which gives us

17 $$ {\overline{w}}_o^{\prime }=\sum {p}_i\left[ t\left({a}_i\right)\left( T-{C}_o- S\right)-{t}^{\prime}\left({a}_i\right)\left(\left(1-{a}_i\right) T+{a}_i\left({C}_o+ S\right)\right)\right]. $$

Referring back to the results for a monomorphic population of owners (see Eq. 7), it becomes apparent that the effect of cautiousness on the evolution of aggressiveness remains unchanged even if there is phenotypic variation in aggressiveness. For evolutionarily stable *t*, the gradient *t* ^′^ is always negative (see Eq. 14):

18 $$ {t}^{\prime }=\frac{- T+{C}_t- S}{2{C}_s} $$

In addition to the direct benefit of defense (first term in the sum in Eq. 17), the evolution of cautiousness in response to variation in aggressiveness therefore results in an added positive selection pressure for higher aggressiveness. Coevolution of defense and cautiousness could in this way greatly stabilize ownership. It is worth noting that cautiousness as well as aggressiveness can be selected for even if defense is never successful in the strict sense, i.e., if it does not reduce the amount of resources an intruder steals (*T* = *S*).

## Simulation

We tested the results of our mathematical analysis in a more detailed individual-based simulation model. We give a short summary of the model that will be expanded on in more detail below: A population composed of a fixed proportion of territorial owners and non-territorial floaters competes for resources that occur in the territories as well as in an unclaimed area accessible to all floaters. During each time step, each floater decides whether to forage either in the unoccupied area (potentially competing with other floaters) or to attempt to intrude into a territory and steal resources. Owners decide whether to start a costly fight in order to attempt to chase away intruders. Fitness of all individuals is determined as sum of resource items foraged minus all fighting costs. For a list of parameters and evolving traits please refer to Table 2.

**Table 2:** Glossary of traits and parameters of the simulation model

Evolving traits	
*a*	Defense probability (owner)
*t*	Intrusion probability (intruder, only in scenarios without sensitivity)
*s*	Sensitivity (intruder, only in scenarios with sensitivity)
*o*	Offset, i.e., intrusion probability at a = 0 (intruder, only in scenarios with sensitivity)
Parameters	
*C*_*t*_	Fighting costs intruder
*C*_*o*_	Fighting costs owner
*C*_*s*_	Costs of sensitivity
*e*	Effectiveness of defense in preventing theft

### Evolution

Following common practice, we assume haploid parthenogenetic individuals with directly heritable traits (i.e., genotype and phenotype are not distinguished). Similar to other studies on resource competition (e.g., Dubois and Giraldeau 2005; Morrell and Kokko 2005), we assume that everything else being equal more successful foraging behavior leads to higher fitness. An individual’s fitness *w*_*i*_ is therefore calculated as overall energy uptake (*u*_*i*_, see below) minus costs through fighting and behavioral flexibility (see below). Population size is fixed, and each generation of individuals completely replaces the previous generation. For each individual in the new generation, a parent from the previous generation is picked with ${p}_{i,\mathrm{prev}}={w}_{i,\mathrm{prev}}/\overline{w_{\mathrm{prev}}}$. An individual’s expected number of offspring is therefore proportional to the individual’s relative fitness. On reproduction, each evolving trait mutates with a probability of 0.01. Mutation step size is normally distributed with a mean of 0 and a standard deviation of 0.1.

### Ecology

At all times, the population consists of 1000 individuals. At the start of the simulation and immediately after reproduction, half of the population is assigned a territory that they will keep for the rest of their life. The remaining individuals become non-territorial floaters. It is important to note that this is purely done as a matter of convenience of implementation and does not imply a specific life history. Since the traits that determine behavior as owner or floater, respectively, are entirely independent, the model is perfectly compatible with more realistic scenarios, for instance, a population where owners and floaters can switch roles but only owners reproduce.

Each unit of space in the common area as well as in the territories is assumed to refill to a level of 1 resource unit at the beginning of each (interaction) time step. The entire habitat has a size of 3000 space units. Territories measure 5 space units while the unoccupied area covers the remaining 500 space units. Unless interrupted (see below), foraging individuals are able to cover 5 space units during one time step. Movements during foraging are assumed to be completely random. If, therefore, several individuals forage in the same area—such as several floaters in the common area or the owner and one or more intruders in a territory—there is a chance that their paths overlap. Given size of the area *A* and space covered by one individual during foraging *f*_*i*_ and taking overlap into account, we can calculate the expected proportion of space that has been visited by at least one individual during the current foraging bout as

19 $$ F=1-\prod_j\left(1-\frac{f_j}{A}\right). $$

All individuals foraging in the same territory or in the common area will have to share the resources contained in that proportion *F*. We assume that an individual’s share is proportional to the space it covers during foraging. With a resource density of 1 unit per space unit, expected resource uptake of an individual *i* is then

20 $$ {u}_i= FA\frac{f_i}{\sum {f}_j}. $$

### Intrusion and fighting

We assume that floaters have a limited home range that covers 20 territories and does not change during their life time. At the beginning of each time step, each floater visits a random territory within its home range and decides whether to attempt to intrude into that territory or whether to forage in the unoccupied area.

In scenarios without sensitivity, floaters intrude with a fixed heritable probability *t*. In scenarios with flexible behavior, individuals adjust their tendency to steal depending on an estimate of the owner’s aggressiveness based on their past experience. For each territory, floaters keep track of the number of times they have been attacked while intruding on that territory *n* ^*a*^ versus the overall number of time steps they spent foraging there *n* ^*f*^. From this the floaters estimate the owner’s attack probability as ${\overset{\sim }{a}}_i={n}_i^a/{n}_i^f$. The adjusted tendency to steal $\overset{\sim }{t}\left(\overset{\sim }{a}\right)$ is then calculated based on trait sensitivity *s* and offset *o* as

21 $$ {\overset{\sim }{t}}_i\left({\overset{\sim }{a}}_i\right)= s{\overset{\sim }{a}}_i+ o $$

A meaningful estimate $\overset{\sim }{a}$ can only be made after a number of attempts to intrude into the same territory. In scenarios with sensitivity, individuals therefore use the sum of *t* and $\overset{\sim }{t}$, weighted by numbers of intrusion *n* ^*f*^ to determine the actual probability *p* to intrude into a territory:

22 $$ {p}_i=\frac{n_i^f{\overset{\sim }{t}}_i+ t}{n_i^f+1} $$

Behavioral complexity can carry a fitness cost (e.g., for maintenance of the required physiology or increased reaction times, see Auld et al. 2010); therefore, floaters pay ${C}_s\left|\overset{\sim }{t}(0)-\overset{\sim }{t}(1)\right|$ energy units per decision.

After all floaters have made their choice, the non-intruding ones move into the common area while the intruders start foraging on the territory they have selected. Owners then decide (according to their trait aggressiveness *a*) for each intruder on their territory whether to attack or not. Attacks result in costly fights (with costs *C*_*i*_ for intruders and *C*_*o*_ for owners, respectively) that are won by the owner and the intruder with equal probability. If the intruder wins, both owner and intruder forage with equal efficiency. If the owner wins, the intruder is chased away and the area it covers during foraging *f* (see Eqs. 19 and 20 above) is reduced to *f* ^*L*^ depending on the effectiveness of defense *e*:

23 $$ {f}_i^L={f}_i\left(1- e\right) $$

Different values of *e* could for example be a result of differences in how quickly owners detect intruders and consequently in how much time intruders have for foraging before being detected. Corresponding to the difference between *T* and *S* in the mathematical model (see Tables 1 and 2), the lower *e*, the less direct benefits defense has for the territory owner.

The presence of several intruders at once is assumed to have no additional effects beyond fighting costs and potential reduction on foraging efficiency (see “Ecology” section).

## Results

Unless mentioned otherwise, all simulations start out with a peaceful (i.e., non-defending), non-cautious ancestral population and run for 20,000 generations. Results are presented as mean values (and standard error) of 10 replicate runs.

### Sensitivity

**Fig. 1:**
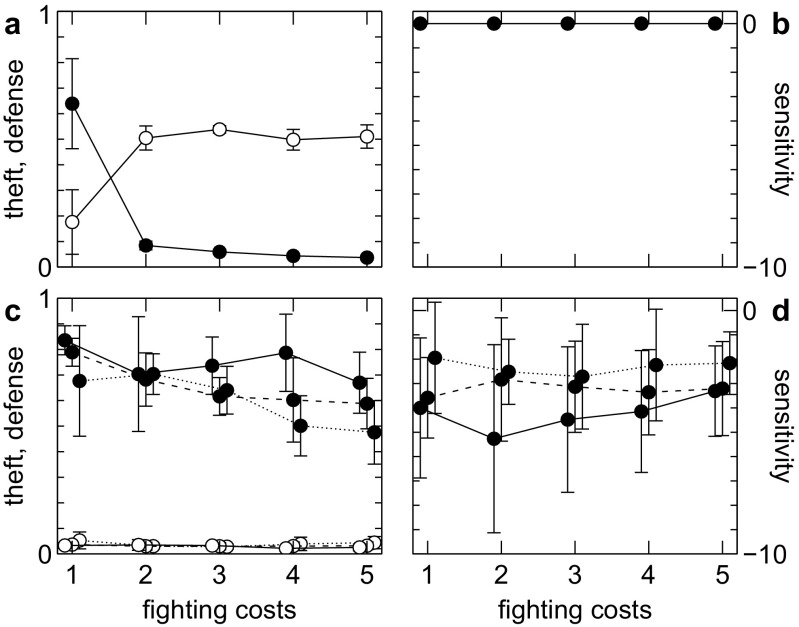
Evolution of ownership at different fighting costs (*c*_*i*_ = *c*_*o*_) with sensitivity set to 0 (**a**, **b**) and evolving (**c**, **d**). Mean and standard deviation (over ten replicate runs) of defense (*left*, *filled circles*), theft (*left*, *lines* with *open circles* at the *bottom* of the graph) and sensitivity (*right*) after 20 k generations are shown for low (*solid line*, *c*_*s*_ = 0), medium (*dashed line*, *c*_*s*_ = 0.05), and high (*dotted line*, *c*_*s*_ = 0.1) costs of sensitivity. Despite effective defense (*e* = 1), ownership only evolves for low fighting costs if there is no sensitivity. If sensitivity can evolve, it does so even for high fighting costs and thus stabilizes ownership

Without sensitivity, ownership only evolves for very low fighting costs for owner and intruder (Fig. 1). For higher costs, defense disappears and theft is high.

If intruders can change their behavior based on perceived aggressiveness of an owner then negative sensitivity evolves (Fig. 1, right). Even a moderate negative slope of tendency to steal versus aggressiveness is sufficient to trigger the evolution of high levels of defense. These in turn lead to very low levels of theft so that ownership becomes established (Fig. 1).

### Ineffective defense

**Fig. 2:**
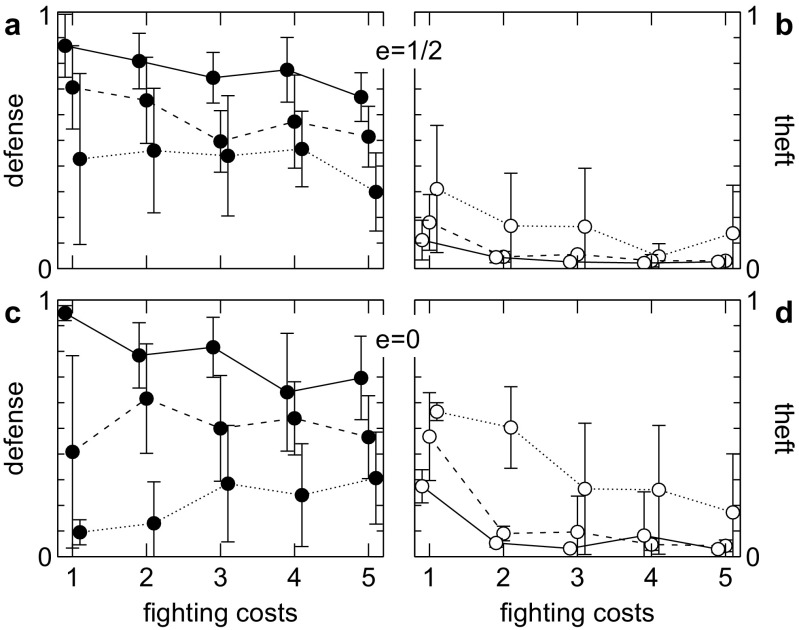
Evolution of ownership at different fighting costs (*c*_*i*_ = *c*_*o*_) for moderately effective (*e* = 1/2, **a**, **b**) and completely ineffective defense (*e* = 0, **c**, **d**). Mean and standard deviation (over ten replicate runs) of defense (*left*) and theft (*right*) after 20 k generations are shown for low (*solid line*, *c*_*s*_ = 0), medium (*dashed line*, *c*_*s*_ = 0.05), and high (*dotted line*, *c*_*s*_ = 0.1) costs of sensitivity. Even if defense has no direct effect, deterrence can be sufficient to maintain ownership

The mathematical analysis predicted that even if defense has little direct effects it can evolve due to the benefits of deterrence. This is confirmed by our simulation. High levels of defense and consequently low levels of theft can even evolve if owners cannot prevent theft at all (Fig. 2).

### Increased variation

Based on the mathematical analysis, we expect that an increased variation in attack probability will cause an increase in selection for cautiousness and thus defense. In order to test this, we added a small proportion (10%) of tough individuals to the population. As owners, these pay only 20% of the fighting costs compared to the rest of the population and consequently can afford to be more aggressive (see Kreps and Wilson 1982).

**Fig. 3:**
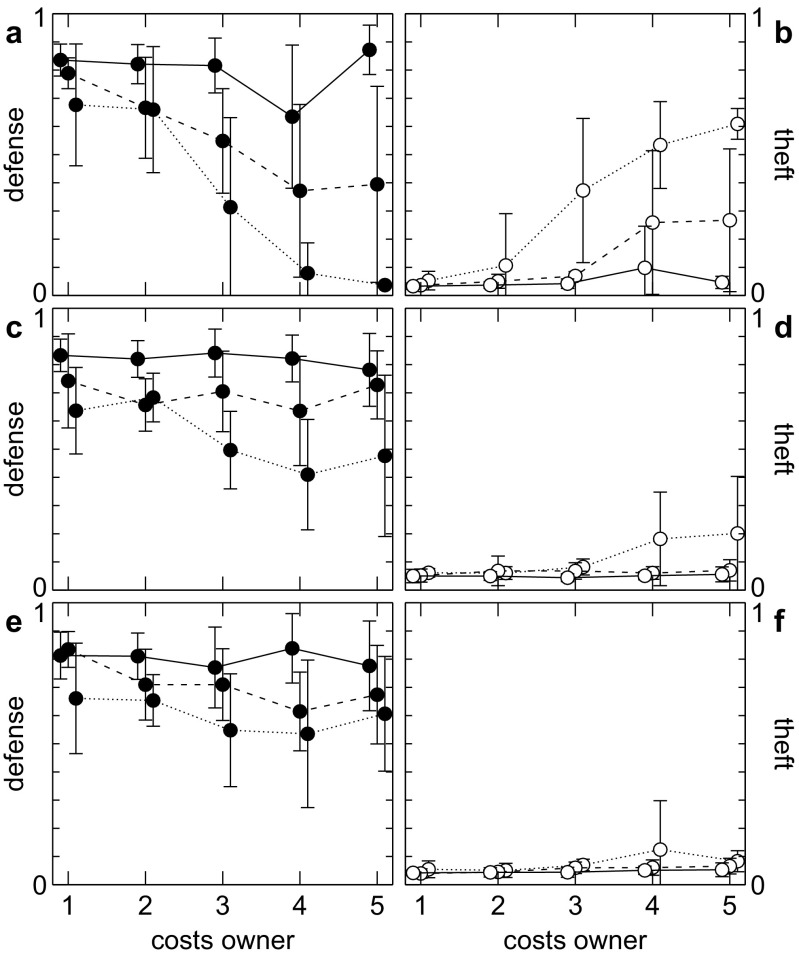
Evolution of ownership at fixed fighting costs for the intruder (*c*_*i*_ = 1) and varying costs of defense. Mean and standard deviation (over ten replicate runs) of defense (*left*) and theft (*right*) after 20 k generations are shown for different costs of sensitivity (low: *solid line*, *c*_*s*_ = 0; medium: *dashed line*, *c*_*s*_ = 0.05; high: *dotted line*, *c*_*s*_ = 0.1). Results are presented for the standard scenario (**a**, **b**), with a small number of tough owners in the population (**c**, **d**) and with intruders choosing the best out of two territories before deciding on intrusion (**e**, **f**). A strong owner disadvantage prevents the evolution of ownership for medium and high costs of sensitivity. Higher variation in *a* as well as additional choice compensate for that [*e* = 1]

As can be seen from Fig. 3, high fighting costs for the owner prevent the evolution of ownership (Fig. 3a, b). The presence of a small number of tough individuals, however, increases the variation in aggressiveness experienced by intruders sufficiently to again let cautiousness and defense coevolve to a point where ownership becomes established (Fig. 3c, d).

### Additional choice

Having floaters choose between one (random) territory and the unoccupied area seems like an artificial restriction given that distances between territories can be small and that individuals have all the information required to make a better decision. We therefore investigated an extension of the model based on the assumption that floaters can choose between intrusion into either of two territories and foraging in the unoccupied area. Each floater is first presented with two random territories (out of its home range). Of those, it picks the one with the higher probability to intrude and only then makes the decision whether to intrude or not.

A comparison of Fig. 3e, f with Fig. 3a, b shows that adding this extra step to the floater’s decision process significantly increases selection for ownership (with higher defense and lower theft) which implies increased selection for sensitivity.

## Discussion

We have shown that if potential thieves or intruders are capable of adapting their behavior to an owner’s aggressiveness in evolutionary time, even small variations in defense propensity can trigger the evolution of increased cautiousness which again strongly selects for higher aggressiveness and thus leads to the establishment of ownership.

In classical models on the evolution of defense of resources (e.g., Maynard Smith and Parker 1976; Houston et al. 1985; Dubois and Giraldeau 2005; Morrell and Kokko 2005; Gintis 2007), ownership is viable since intrusion or theft is uneconomical. This is due to the fact that the expected gain from a conflict with the owner (i.e., probability to win times value of resource) is lower than the fighting costs. Although this can be interpreted as a deterrence effect of defense (in particular in models with optional fights, e.g., Dubois and Giraldeau 2005), it takes place entirely in evolutionary time and has therefore no bearing on the fitness benefits of defense itself. Consequently, defense in these models cannot be selected for if it has no immediate effect on the intruder’s chances of success (Selten 1978). Similar to Switzer et al. (2001), our results show that cautiousness, i.e., flexible reactions to the owner’s aggressiveness, leads to deterrence in sub-evolutionary time which can increase the benefits of defense up to the point where direct effects are no longer necessary.

Furthermore, neither cautiousness nor defense has to be assumed to pre-exist in the population—small random variations are sufficient to trigger a positive feedback between the two traits that leads to the establishment of defense. Defense and ownership can therefore be evolutionarily stable and even emerge in populations that would remain entirely peaceful in the absence of deterrence. This suggests that the feedback between cautiousness and defense can play a much greater role in stabilizing ownership than the immediate effect of defense.

Our results can therefore give a possible explanation for the existence of property in situations where the resource in question is not strictly defendable in the classical sense. Hinsch and Komdeur (2010), for example, predicted that in many situations territory owners should profit from poaching on their neighbor’s territory to such a degree that territory defense would become untenable in the long term. The existence of deterrence could explain why even in cases where resources would be easily accessible to neighbors only low levels of poaching occur and territoriality is maintained (Carpenter and MacMillen 1976; Young and Monfort 2009; Dantzer et al. 2012).

If defense has no immediate benefit, it becomes functionally equivalent to punishment (Raihani et al. 2012). In studies on the evolution of cooperation, punishment of cheaters has been proposed as a way that the benefit of unilateral non-cooperation is sufficiently reduced for altruistic behavior to become advantageous in comparison. However, since punishment is usually assumed to be costly it can—in equivalence to ineffective defense—only be selected for if it has some additional positive effects for the punisher (Gardner and West 2004; Schoenmakers et al. 2014). Similar to our results, it has been shown that the availability of knowledge (by reputation or experience) about the individuals’ willingness to punish combined with the ability to react to this information can lead to a deterrence effect that is sufficient to compensate for the costs of punishment (Sigmund et al. 2001; dos Santos et al. 2010; Thompson et al. 2014). Together with our results, this demonstrates that punishment and defense can be seen as two points on the same continuum.

The occurrence of the described feedback effect in our model rests on a number of conditions concerning physiology and ecology of the modeled species. The generality of our results is determined by how likely it is that these conditions are met in natural populations.

First, individuals have to be able to obtain information about the aggressiveness of their competitors either by personal experience through repeat interaction or through other mechanisms such as direct observation, reputation, or signals (but see Hurd 2006). In any species with either stable social groups or a stable spatial organization, this condition will be naturally met (Earley 2010).

Second, they have to possess the cognitive capabilities to store and use this information. Most vertebrates as well as many invertebrate species are assumed to be capable of at least simple forms of learning (Brembs 2003). Basic operant conditioning in combination with either spatial memory or individual recognition should be sufficient for the type of information processing assumed in our model (see Gutnisky and Zanutto 2004; Tanabe and Masuda 2011).

Third, there has to be sufficient variation in aggressiveness or attack rate to trigger the feedback. While mutation rates in our simulation are relatively high, epigenetic effects as well as environmental stochasticity during development provide additional sources of variation in reality that were not included in the model (see McNamara et al. 2008; Wolf et al. 2008). Our results furthermore suggest that all variations between individuals that lead to variations in attack rate can serve as trigger for the evolution of cautiousness. Besides purely genetic or physiological effects therefore all variation that either produces a phenomenological variation in attack rate (such as detection probability due to, e.g., habitat differences) or consistently induces different strategic decisions in different individuals (such as territory quality, individual size, condition, or experience) will have the same effect (see Kreps and Wilson 1982; Przepiorka and Diekmann 2013). It seems reasonable to assume that at least some of these sources of variation will be present in most natural populations.

It is also important to note that while at least implicitly our models suggest scenarios with intraspecific competition, there is no intrinsic reason to assume that the same mechanism could not apply to the interaction between individuals of different species such as interspecific kleptoparasitism (Iyengar 2008).

In conclusion, we think that the conditions for an evolutionary feedback between cautiousness and defense are probably met in many populations in which defense of property occurs. This has ramifications for empirical as well as theoretical research. In empirical studies, great effort has been invested to determine the costs and benefits of defense. If a large part of the adaptive value of defense however consists in scaring away competitors from challenging the owner in the first place, the measured benefits will necessarily be too low. In most previous models on defense, e.g., in the context of mate guarding, territoriality, resource defense, or kleptoparasitism, only direct benefits of defense have been investigated thereby likely significantly underestimating the range of parameter values for which defense and with it ownership can be evolutionarily stable.

Finally, we want to note that a model can only ever be a proof of principle. Whether the mechanism we propose does in fact play a role in a given system can therefore only be determined with the help of empirical research.

## Data availability

The source code of the simulation program as well as the generated data is available in the figshare repository https://doi.org/10.6084/m9.figshare.4959947 or from the authors on request.

## Appendix

### Selection for sensitivity

Using the fitness function from the main text (Eq. 11)

24 $$ {w}_t=-{c}_s(t)+\sum {p}_i\left[\left(1- t\left({a}_i\right)\right)\widehat{P}+ t\left({a}_i\right){I}_i\right], $$

and assuming that a mutant differs in tendency to steal by a small amount *s*_*i*_, we get for the fitness of the mutant

25 $$ {w}_{t, m}=-{c}_s\left( t+ s\right)+\sum {p}_i\left[\left(1-\left(\widehat{t}\left({a}_i\right)+{s}_i\right)\right)\widehat{P}+\left(\widehat{t}\left({a}_i\right)+{s}_i\right){I}_i\right]. $$

The fitness difference between mutant and residents then resolves to

26 $$ \begin{array}{lll}{w}_{t, m}-{w}_t& =& -{c}_s\left( t+ s\right)+\sum {p}_i\left[\right(1-\left(\hat{t}\right({a}_i\left)+{s}_i\right)\left)\hat{P}+\right(\hat{t}\left({a}_i\right)+{s}_i\left){I}_i\right]+\\ {}& & {C}_s(t)-\sum {p}_i\left[\right(1-\hat{t}\left({a}_i\right)\left)\hat{P}+\hat{t}\right({a}_i\left){I}_i\right].\end{array} $$

This can be simplified as

27 $$ \begin{array}{lll}\varDelta {w}_t& =& -\varDelta {c}_s+\sum {p}_i\left(-{s}_i\hat{P}+{s}_i{I}_i\right)\\ {}& =& -\varDelta {c}_s+\sum {p}_i{s}_i\left({I}_i-\hat{P}\right).\end{array} $$

As mentioned in the main text, we now assume the costs of sensitivity to be proportional to the variance of the values of *t* (as realized in a given resident population with its associated distribution of aggressiveness *a*). If we define *t*_*i*_ ≔ *t*(*a*_*i*_), we can write costs of sensitivity as

28 $$ {c}_s(t)={C}_s VAR\left({t}_i\right) $$

From this, we can derive the change in costs:

29 $$ \begin{array}{cccc}\varDelta {c}_s& =& & {C}_s\left(\mathrm{VAR}\left(\hat{t}+ s\right)-\mathrm{VAR}\right(\hat{t}\left)\right)\kern1em \\ {}\kern.5em & =& \kern1em & {C}_s\left(\mathrm{VAR}\left(\hat{t}\right)+\sum {p}_i{s}_i\right(2\left({\hat{t}}_i- E\hat{t}\right)+{s}_i\left(1-{p}_i\right)\left)-\mathrm{VAR}\right(\hat{t}\left)\right)\kern1em \\ {}\kern1em & =& \kern1em & {C}_s\sum {p}_i{s}_i\left(2\left({\hat{t}}_i- E\hat{t}\right)+{s}_i\right(1-{p}_i\left)\right).\kern1em \end{array} $$

Since we assume small mutation steps (*s*_*i*_ ≪ *t*_*i*_), the last term in the sum can be neglected, leaving

30 $$ \varDelta {c}_s={C}_s\sum {p}_i{s}_i2\left({\widehat{t}}_i- E\widehat{t}\right). $$

Plugging this into *Δw* (Eq. 27), we obtain

31 $$ \varDelta {w}_t=\sum {p}_i{s}_i\left({I}_i-\widehat{P}-2{C}_s\left({\widehat{t}}_i- E\widehat{t}\right)\right). $$

For the sake of convenience (and without loss of generality with respect to evolutionary dynamics), we rescale all payoffs by costs of sensitivity. We obtain scaled fitness as *w*′_*t*_ ≔ *w*_*t*_/2*C*_*s*_ and the scaled benefit of the outside option as ${\widehat{P}}^{\prime}\coloneq \widehat{P}/2{C}_s$. We also define the expected (rescaled) payoff of intrusion as

32 $$ {I}_i^{\prime }:={I}_i/2{C}_s. $$

This gives us a simplified expression:

33 $$ \varDelta {w}_t^{\prime }=\sum {p}_i{s}_i\left({I}_i^{\prime }-{\hat{P}}^{\prime }-\left({\hat{t}}_i- E\hat{t}\right)\right). $$

We can now see that if

34 $$ {I}_i^{\prime }-{\hat{P}}^{\prime }={\hat{t}}_i- E\hat{t}\kern0.70em \forall i $$

then invasion fitness will always be 0 for any combination of *s*_*i*_. No mutant can therefore spread in the resident population which makes this an evolutionarily stable strategy.

On the other hand, if we assume a population with a few “mismatched” *t*_*i*_, so that

35 $$ {I}_j^{\prime }-{\hat{P}}^{\prime }>{\hat{t}}_j- E\hat{t}, j\in J $$

and

36 $$ {I}_k^{\prime }-{\hat{P}}^{\prime }<{\hat{t}}_k- E\hat{t}, k\in K, $$

then in particular any mutant with *s*_*j*_ > 0 and *s*_*k*_ < 0 (∀ *j* , *k*) will have a positive invasion fitness and thus will be able to invade, thereby fulfilling the condition for convergence stability.

For a given *a*, Eq. 34 therefore defines a unique evolutionarily (and convergence) stable strategy *t* ^*^:

37 $$ {t}_i^{\ast }={I_i}^{\prime }-{\widehat{P}}^{\prime }+ E{t}^{\ast } $$

Taking the expected value of both sides, we obtain

38 $$ E\left({t}_i^{\ast }- E{t}^{\ast}\right)= E{I_i}^{\prime }-{\widehat{P}}^{\prime }, $$

which gives us

39 $$ {EI}^{\prime }={\widehat{P}}^{\prime }. $$

With this and using $\widehat{P}= P(Et)$, we can now derive an explicit solution for *t* that only depends on *a*:

40 $$ \begin{array}{lll}{t}_i^{\ast }& =& {I}_i^{\prime }-\mathrm{E}{\mathrm{I}}^{\prime }+{P}^{-1}\left(\mathrm{EI}\right)\\ {}& =& \frac{I_i-\mathrm{E}\mathrm{I}}{2{C}_s}+{P}^{-1}\left(\mathrm{EI}\right)\end{array} $$
